# Dietary Quality in Bipolar Disorder Compared to Unipolar Depression (Current and Remitted) and Healthy Controls: The Netherlands Study of Depression and Anxiety

**DOI:** 10.1111/bdi.70104

**Published:** 2026-03-17

**Authors:** M. A. Riedinger, M. Koenders, H. W. Jeuring, M. L. Molendijk, B. W. J. H. Penninx, N. J. A. van der Wee, M. de Leeuw, E. J. Giltay

**Affiliations:** ^1^ Department of Psychiatry Leiden University Medical Centre Leiden the Netherlands; ^2^ Psychiatric Institute, GGZ Rivierduinen Outpatient Clinic for Mental Disability and Psychiatry Leiden the Netherlands; ^3^ Faculty of Social Sciences, Leiden University Institute of Psychology Leiden the Netherlands; ^4^ Department of Psychiatry, University Medical Center Groningen University of Groningen Groningen the Netherlands; ^5^ Institute of Psychology, Clinical Psychology Unit Leiden University Leiden the Netherlands; ^6^ Institute for Brain and Cognition Leiden University Medical Center Leiden the Netherlands; ^7^ Department of Psychiatry, Amsterdam UMC, Vrije Universiteit Amsterdam the Netherlands; ^8^ Psychiatric Institute, GGZ Rivierduinen Bipolar Disorder Outpatient Clinic Leiden the Netherlands; ^9^ Health Campus the Hague, Department of Public Health & Primary Care, Leiden University Medical Center the Netherlands

**Keywords:** bipolar disorder, depressive disorder, diet, dietary quality, major depressive disorder

## Abstract

**Background:**

Patients with bipolar disorder (BD) have an increased risk to develop cardiovascular disease. Western diets have been hypothesized to increase the risk of cardiovascular disease in BD, but dietary habits in BD have not been extensively studied. We therefore assessed in a large cohort dietary quality in BD patients, in patients with current and remitted unipolar depression (UD), and healthy controls (HC).

**Methods:**

In total 1358 participants were included from the Netherlands Study of Depression and Anxiety (NESDA) and categorized into four groups: BD (*n* = 100, 48.0% male, mean age 50.9), current UD (*n* = 199, 28.0% male, mean age 52.4), remitted UD (*n* = 722, 29.8% male, mean age 52.4), and HC (*n* = 337, 40.7% male, mean age 51.2). Diet was assessed through the 238‐item Food Frequency Questionnaire (FFQ), which yielded the ‘Mediterranean Diet Score’ (MDS). Dietary scores were compared using multivariate regression analyzes adjusting for sociodemographics, physical activity, and smoking.

**Results:**

BD patients scored significantly lower on the MDS than those with remitted UD (*p* = 0.01) and healthy controls (*p* = 0.02) but did not differ from those with current UD. Effect sizes were 0.24 for BD vs. remitted UD and 0.25 for BD vs. HC. Furthermore, BD patients had on average a higher waist circumference (*p* = 0.03) and BMI (*p* = 0.02) than healthy controls.

**Conclusion:**

The average dietary quality of BD patients was of lesser quality compared to that in patients with remitted UD and HC. This may have contributed to the increased waist circumference and higher BMI we found among BD patients, with its adverse health consequences.

## 1 Introduction

Bipolar disorder (BD) is a psychiatric disorder affecting approximately 1%–2% of the world population [1]. BD is characterized by cycles of depression and (hypo)mania, and associated with a significant reduction in overall quality and length of life, functioning, and physical health. Apart from suicide, which lowers life expectancy of patients with BD, the increased prevalence of comorbid cardiovascular disease (CVD), obesity, and diabetes mellitus further contributes to the poor overall physical health of patients with BD [1, 2]. BD has been associated with an unhealthy lifestyle, including a higher incidence of substance use (smoking and alcohol consumption), lower physical activity, and poor adherence to medical treatment. Side effects of psychotropic medication were partially to blame for weight gain and dyslipidemia [3, 4]. Dietary habits are a particular important lifestyle factor in relation to the risk of obesity, diabetes, and CVD, [3] and can ameliorate these risks. However, there is currently only limited data available on dietary quality c.q. habits in patients with BD.

The fluctuating mood states of BD may pose challenges for patients in maintaining a healthy diet. During depressive states, individuals may experience low energy levels and loss of initiative, often resulting in not planning or cooking meals, but choosing easy, energy‐dense snacks more often [5]. Changes in appetite, particularly in cases of atypical depression, can lead to unhealthier choices with craving for salty and fatty foods [6]. Furthermore, the loss of energy inhibits patients from engaging in physical activity. The sedative effects of psychotropic medications and increased caloric intake—possibly due to enhanced feeling of hunger and a reduced sense of fullness caused by several antipsychotics and mood stabilizers [7]‐ may further shift the balance toward an inactive lifestyle and unhealthy diet. Moreover, many antipsychotics, which are often used in people with BD [8], have metabolic side effects, inducing dyslipidemia, which can further complicate the management of cardiovascular health [7, 9, 10]. In line with this, several meta‐analyzes have found a strong association between BD and obesity, showing that individuals with BD are more often obese than controls [11, 12, 13].

Although weight gain and obesity are important health issues in BD, the role of dietary habits in BD has not been extensively studied [14]. Previous research summarized in a meta‐analysis has indicated that the diet of individuals with psychiatric disorders in general tends to be less healthy than that of their counterparts without such disorders [2]. Specifically, five previous studies have examined the dietary quality of BD patients, including between 23 and 1945 patients [3, 15, 16, 17, 18]. Of these studies, four also included healthy control groups [3, 15, 16, 17], while one study compared diet scores to those of the general public [18]. The largest study did not show a significant difference in the average intake of vegetables or fruit [15]. In four other studies, BD patients consumed on average more calories and sugar, averaging a higher glycemic load than healthy controls, and adhered more often to a more Western‐style dietary pattern [3, 16, 17, 18]. Furthermore, waist circumference and fasting triglycerides were significantly higher in euthymic BD patients when compared to healthy controls [17]. A cross‐sectional assessment of diet in a cohort of 732 BD patients revealed that their Rapid Eating Assessment for Participants—shortened version scores were lower than the mean score of the general public in the U.S., and that an unhealthier diet was associated with higher cardiovascular risk factors [18]. However, there is a lack of studies that used validated Food Frequency Questionnaire (FFQ) data [19], with only two previous studies using an extensive FFQ [16, 17] and one using a 24‐h dietary recall [3] while the other studies relied only on questionnaires of only 16 items or less [15, 18].

Compared to BD, there is much more knowledge on diet in patients suffering from unipolar depression (UD). Research has consistently shown that the diet of persons suffering from UD tends to be less healthy than that of healthy controls [2]. Several meta‐analyzes have investigated the relationship between diet quality and depressive symptoms, with most of them concluding that patients adhering to the Mediterranean diet had less severe symptoms of depression [20] and that severity of depressive symptoms was inversely associated with diet scores [21]. However, these meta‐analyzes caution that causality is difficult to assess since they included mostly observational longitudinal studies, and not all included studies adjusted for known confounders. Importantly, studies focusing specifically on BD are rare, while the burden of disease is high and due to the recurrent nature of mood episodes, persons with BD typically suffer from multiple episodes during their life.

In summary, only a few studies on diet quality have focused on BD specifically. In the longitudinal Netherlands Study of Depression and Anxiety (NESDA), diet quality was assessed with the validated and extensive 238‐item FFQ in a large group of individuals with and without mood disorders. In this cohort, persons suffering from current UD had less healthy diets than healthy controls [22], but BD was not examined. The aim of the current study is to compare the diet and, secondly, other covariates of cardiovascular disease of individuals with BD versus individuals with UD (both current and remitted), and healthy controls. Considering the recurrent depressive episodes commonly found in BD, we hypothesized that individuals with BD consume, on average, poorer quality diets than healthy controls and individuals with remitted UD. Secondly, we hypothesized that diet quality would not be different from that in individuals with current UD since lower diet quality was reported in patients suffering from current UD compared to healthy controls in previous studies.

## 2 Participants and Methods

### 2.1 Participants

Data were gathered from participants of the NESDA study [23]. A total of 2069 participants participated in wave 6, at which the FFQ was assessed, which corresponds to 69.4% of the original 2981 at baseline cohort. A total of 1671 (80.7% of wave 6 respondents) participants provided a completed FFQ, which yielded dietary quality scores and of whom 1358 (81.3%) were included in the analysis. Participants were excluded when they had a current Composite International Diagnostic Interview (CIDI) based anxiety disorder at wave 6, without a comorbid depressive disorder. The participants who were included were divided into four groups based on CIDI diagnoses; one group consisted of patients who were diagnosed with BD during the course of the study, using the CIDI (*n* = 100; male 48%, mean age 50.9 ± 12.2); 58 patients were diagnosed with BD in wave 3 (2 years after baseline), 17 more at wave 4 (4 years after baseline), 19 at wave 5 (6 years after baseline), and six more were identified by the time of wave 6 (9 years after baseline). The second group consisted of patients with CIDI‐based current UD at the time of the FFQ measurement or with a recent diagnosis (< 6 months) of dysthymia (*n* = 199; male 28.0%, mean age 52.4 ± 11.8). The third group had remitted UD (*n* = 722; male 29.8%, mean age 52.4 ± 13.0). The remaining participants were healthy controls (*n* = 337; male 40.7%, mean age 51.2 ± 14.6). Participants were interviewed at wave 6, 9 years after the baseline measurement. The study was conducted in compliance with the Declaration of Helsinki and approved by a central medical ethics committee (Amsterdam University Medical Centre, The Netherlands) and by the local committees of the other sites (Leiden, Groningen). Written informed consent was obtained from all participants.

### 2.2 Dietary Quality Data

A 238‐item FFQ, an extended version of a reproducible and biomarker validated FFQ [24, 25, 26], yielded dietary data for all included participants. From this FFQ, which assessed the usual diet over 4 weeks, the Mediterranean Diet Score (MDS) could be obtained. The Mediterranean Diet Score (MDS) [27] is calculated by assessing the weekly intake of 11 different food components: non‐refined cereals, vegetables, fruits, legumes, potatoes, fish, meats, poultry and dairy products (full fat), as well as olive oils and alcohol. These food groups were scored on a scale from 0 to 5 points, with 0 points meaning no intake and the scores of 1–5 describing different ranges of intake. These 11 food components, were also used as subscores in the explorative analyzes. Alcohol, meat products, poultry and full fat dairy products were reversely scored, leading to a maximum score of 5 points when these components are rarely or never consumed. This score, with a range from 0–55, has been found to be predictive of cardiovascular morbidity (e.g., dyslipidemia, hypertension, diabetes mellitus and CVD), with lower scores associated with an increased risk.

### 2.3 Mental Health Assessment

The Composite International Diagnostic Interview (CIDI) [28] is a clinical instrument assessing the presence of possible psychiatric diagnoses. The outcome of the subset questionnaire on BD was used to determine whether a participant had been diagnosed with BD during the study. The subset for anxiety disorders was used to exclude patients with current anxiety disorders (generalized anxiety disorder, social phobia, panic disorder, agoraphobia), without current UD. The subset questionnaire on depressive disorder was used to differentiate between current UD (major depressive disorder (MDD) and dysthymia) in the past 6 months, and remitted UD.

The Mood Disorder Questionnaire, Dutch language (MDQ‐NL) [29] is a screening questionnaire of 15 questions assessing possible (hypo)manic symptoms in persons who have (had) a depressive episode. In this study, the total out of all thirteen possible symptoms was assessed.

The Inventory of Depressive Symptoms (IDS) is a 30‐item scale which assesses depressive symptoms and covers all nine dimensions on which a DSM IV depressive episode can be diagnosed. A score below 14 points signifies no depressive episode. The ranges of severity scores can be categorized as follows: 14–25 mild depressive symptoms, 26–38 moderate depressive symptoms, 39–48 severe depressive symptoms, and 49 points or above are categorized as very severe depressive symptoms. The internal validity of this questionnaire is high (Cronbach's alpha 0.94) [30]. Table S1 shows current depressive symptom severity (IDS‐SR score) and lifetime (hypo)mania severity (sum of MDQ positive items) for the cohort.

### 2.4 Covariates and Predictors of General Health and Cardiovascular Risk

All participants completed questionnaires on the current use of medication, which was coded using the Anatomical Therapeutic Chemical (ATC) classification system. Medication use was subdivided into frequent use (50% or more of the time) or no use.

Furthermore, weight and height were measured at the study visit, yielding the Body Mass Index (BMI). Waist and hip circumferences were also measured, yielding the waist‐to‐hip ratio (WHR) next to the individual measurements. Other patient characteristics such as educational level, age, smoking status (yes/no), and alcohol intake according to Alcohol Use Disorders Identification test (AUDIT) [31] were also assessed at the study visit. Since alcohol intake is also part of the MDS score, the AUDIT score was only used in analyzes that did not include the MDS. If any of these possible covariates were missing, the most recent available value from a preceding wave for this covariate was imputed. This was the case for 168 BMI measurements, 172 waist and hip measurements, 103 activity scores, 27 alcohol consumption measurements, and 15 IDS scores.

### 2.5 Statistical Analysis

Sociodemographic characteristics were presented per group as number (with %) or mean (with standard deviation [SD]), when appropriate. ANOVA testing was used for continuous variables and chi‐squared tests for dichotomous variables (See Table 1), for testing difference compared to the BD group.

**TABLE 1:** Basic characteristics of NESDA participants (*n* = 1358), divided into groups by diagnosis.

	Lifetime BD (*n* = 100)	Current UD (*n* = 199)	Remitted UD (*n* = 722)	Healthy controls (*n* = 337)
Sociodemographic characteristics				
Male sex, *n* (%)	48 (48)	56 (28.1)*	215 (29.8)*	137 (40.7)
Age at wave 6, years, mean (SD)	50.9 (12.2)	52.4 (11.8)	52.4 (13.0)	51.2 (14.6)
Level of education:				
Basic	8 (8.0)	10 (5.0)	25 (3.5)	6 (1.8)
Intermediate	55 (55.0)	95 (47.7)*	355 (49.2)*	126 (37.4)*
Higher	37 (37.0)	94 (47.2)	342 (47.3)	205 (60.8)
Lifestyle factors				
Current smoking, *n* (%)	36 (36.0)	45 (22.6)*	192 (26.6)	47 (13.9)*
BMI; mean(SD)	27.3 (4.8)	26.9 (5.2)	26.3 (4.9)*	25.6 (4.7)*
Waist circumference in cm; mean, SD	96.7 (14.0)	94.0 (14.6)	92.2 (14.1)*	92.0 (14.0)*
Waist to Hip ratio, mean, SD	0.91 (0.09)	0.89 (0.09)*	0.88 (0.09)*	0.89 (0.10)*
Hazardous drinking, *n* (%)	28 (28)	61 (30.7)	166 (23.0)	40 (11.9)*
Physical activity				
Low	26 (26)	56 (28.1)	134 (18.6)	64 (19)
Intermediate	40 (40)	80 (40.2)	313 (43.4)	145 (43)
High	34 (34)	63 (31.7)	275 (38.1)	128 (38)
Clinical characteristics				
MDQ‐NL number of ‘yes’ out of 13 at wave 6; mean (SD)	4.52 (3.6)	2.58 (2.5)*	1.78 (2.4)*	0.84 (1.7)*
IDS‐SR, mean (SD)	24.6 (11.9)	28.0 (12.5)	13.1 (8.8)*	6.1 (5.2)*
Number of chronic diseases mean, (SD)	1.11 (1.1)	0.92 (1.0)	0.82 (1.0)*	0.61 (0.9)*
Medication (frequent) use wave 6:				
Tricyclic antidepressants *n*, (%)	7 (7.0)	15 (7.5)	26 (3.6)	0*
SSRI's *n*, (%)	27 (27.0)	47 (23.6)	83 (11.5)*	0*
Other antidepressants *n*, (%)	14 (14.0)	24 (12.1)	38 (5.2)*	0*
Total antidepressant *n*, (%)	44 (44.0)	80 (40.2)	146 (20.2)*	0*
Antipsychotics *n*, (%)	10 (10.0)	12 (6.0)*	6 (0.8)*	0*
Benzodiazepine *n*, (%)	9 (9.0)	14 (7.0)	21 (2.9)*	0*
Anti‐epileptic medication *n*, (%)	10 (10.0)	8 (4.0)*	12 (1.7)*	4 (1.2)*

*Note:* IDS‐SR: All variables were tested for significant difference between lifetime BD and the three other groups (significant values *p* < 0.05). Inventory of Depressive Symptoms – self rating, total score. MDQyes: amount of confirmative answers out of 13 of the Mood Disorder questionnaire. Age at moment of interview and education measured in years. Hazardous drinking was assessed using the Alcohol Use Disorders Identification test (AUDIT); a score of 8 or higher indicating hazardous drinking [31]. Values in the same row with * are significantly different from the BD lifetime group, *p* < 0.05 (post hoc test). For activity levels as well as level of education, the post hoc test was done over all categories. Missing values after imputation of most recently known value: MDQyes total missing: 17, number of chronic diseases total missing: 49. For all types of medication, frequent use (daily or > 50% of the time) is depicted. Infrequent use was in total antiepileptic medication 3 (one patients in BD, current and remitted UD respectively), tricyclic antidepressants 1 (current UD), SSRI's 1 (remitted UD), antidepressants total 4 (2 patients in both current and remitted UD), other antidepressants 2 (one patients in both current and remitted UD group) benzodiazepines: 12 for BD, 22 for current UD, 56 for remitted UD, 3 for HC.

All (e.g., MDS total score and subscores) scores were standardized before their statistical analyzes and in subsequent analyzes, to enable direct comparison of effect sizes as differences in the units of measurement did not affect the analysis. Different ANCOVA models were used to detect differences between groups, with post hoc comparisons at an 0.05 alpha level. After using the crude model, model 1 adjusted for age, sex and level of education. Model 2 additionally adjusted for smoking and physical activity. Influence of lifetime manic symptoms (MDQ yes answers) was tested by regression analysis on the whole cohort, adjusted for all previously mentioned confounders. Differences between the four groups in other covariates (BMI, waist circumference and Waist‐to‐Hip ratio) were tested using additional adjustment for alcohol and subsequently for use of antipsychotic medication. These analyzes were done in all included participants combined, as well as separately for men and women. Explorative regression analysis was performed using standardized scores of depressive symptoms and lifetime (hypo)mania severity, while adjusting for main confounders as described in model 1. All analyzes were done using SPSS version 29.0.0 and R (version 4.2.2. [2016]) [32] was used to create forest plots using the ‘forestplot’ package.

## 3 Results

In comparison to patients with remitted UD and current UD, BD patients were more often male (*p* < 0.001 for both comparisons), while the number of males did not differ between healthy controls and BD patients. As expected, BD patients scored significantly higher on the MDQ‐NL than all three other groups (all characteristics are summarized in Table 1). IDS‐SR did not differ significantly between current UD and BD, but scores were significantly higher for BD when compared to remitted UD patients and healthy controls. BD patients were similar in their usage of antidepressants (SSRI, TCA and other) and benzodiazepines to current UD patients, but more often used antipsychotics (10% vs. 6%). Patients with BD were more likely to use any type of psychotropic medication relative to patients with UD in remission, except for tricyclic antidepressants, and, as expected, more likely to use any type of psychotropic medication compared to healthy controls.

### 3.1 Mediterranean Diet Score (MDS)

**FIGURE 1:**
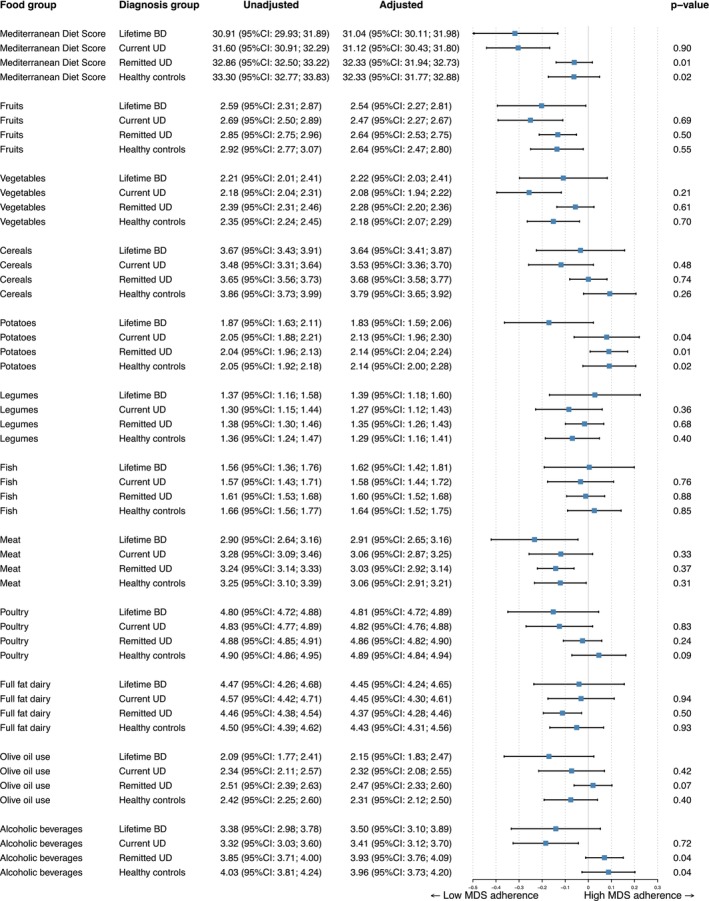
Total MDS score and subscores of BD patients compared to scores of patients with current UD, remitted UD, and healthy controls. Crude and adjusted differences of total and subscore by ANCOVA. The adjusted mean difference is the result of adjustment for age, sex, educational level, physical activity, and smoking (Model 2) *p*‐values shown are *p*‐values compared to BD group after adjustment for confounders.

Lifetime BD patients scored significantly lower on average on the MDS than patients with remitted UD and healthy controls in the crude model (unadjusted means of MDS in Figure 1). These differences persisted after first adjusting for age, sex and educational level (model 1: (standardized adjusted mean difference of BD compared to remitted UD; −0.293 95% CI:−0.493; −0.092 *p* = 0.004) and BD compared to healthy controls (standardized adj. mean diff.: −0.323 95% CI: −0.538; −0.109, *p* = 0.003)) and additional adjustment for physical activity and smoking (model 2: standardized adjusted mean difference of BD to remitted UD; −0.257 95% CI: −0.455; −0.058 *p* = 0.011) and BD to healthy controls (standardized adj. diff.: −0.257 95% CI: −0.470; −0.043, *p* = 0.018) (see Figure 1). The effect sizes for these differences were small (Cohen's d = 0.24 for the difference between BD to current UD and d = 0.25 for the difference between BD and healthy controls). Significant differences in scores were not found between BD and current UD patients in any of the statistical models. Table S2 presents the association between current depressive symptom severity (IDS‐SR score) and lifetime (hypo)mania severity (sum of MDQ positive items) in relation to diet quality, shown separately for participants with BD and for all participants combined (including those with current UD). Explorative analyzes showed that neither IDS‐SR scores, nor lifetime MDQ scores were associated with the diet quality within the BD group (*n* = 97). In the complete cohort (*n* = 1340), IDS scores were associated with poorer dietary quality (standardized beta: −0.151, 0 = 0.001).

### 3.2 Components of the Mediterranean Diet Score

Explorative analyzes were done to assess the driving components of the difference in MDS scores (see Figure 1). Four out of eleven components showed an unfavorable trend for patients with BD compared to healthy controls: Fruit, meat, poultry, olive oil use were all leaning toward the lower scores for the BD patients. Two additional components were statistically significantly poorer for BD patients compared to healthy controls after adjustment for confounders (model 2): potatoes (*p* = 0.02) and alcoholic beverages (*p* = 0.04). Patients with remitted UD seemed to follow the trend of healthy controls and patients with current UD scored closer to patients with BD.

### 3.3 Antropometrics: BMI And Waist Circumference Differences

Average BMI differences among subgroups were analyzed using a model which adjusted for sociodemographic factors (model 2) as well as alcohol intake and the use of antipsychotics. There was a significant difference between patients with BD and healthy controls (respective mean values: 27.3 kg/m^2^ vs. 25.6 kg/m^2^, mean difference 1.31 95% CI 0.22; 2.4, *p* = 0.02). WHR did not differ significantly between BD and any of the comparison groups using the same model. Also, no significant differences were found when men and women were analyzed separately. Patients with BD had a significantly larger average waist circumference than patients with remitted UD and healthy controls after adjustment for all previously mentioned covariates, except for antipsychotic medication (mean differences respectively to remitted UD and healthy controls: 2.66 cm 95% CI 0.14; 5.3, *p* = 0.049 and 3.1 cm, 95% CI 6.0; 0.28, *p* = 0.03). A similar effect size was found after the additional adjustment for the use of antipsychotic medication, but differences were no longer statistically significant. There was no significant difference in effect size between sexes, tested with the interaction term between sex and group.

## 4 Discussion

The main finding of this study was that patients with BD consumed on average less healthy diets when scored on the MDS than patients with remitted UD and healthy controls, but did not differ from that of patients with current UD. The MDS differences could not be attributed to a single cause, but rather to a combination of factors, such as more frequent alcohol abstinence and higher intakes of meat and poultry, and a lower intake of fruit, potatoes, cereals, and olive oil in patients with BD compared to patients with remitted UD and healthy controls. Thus, our findings show an overall less healthy diet in patients with BD. Although the effect sizes are small, similar to those in the previous meta‐analysis that assessed diet in current UD compared to healthy controls [21], the implications are relevant. Improving diets of BD patients could lead to meaningful health benefits on the population level and should be considered as part of the comprehensive treatment plan for BD.

When compared to previous studies, the data reported here align with the existing literature, but offer a slightly more nuanced view on the diets of BD patients. Five previous studies also found less healthy diets in BD patients [3, 15, 16, 17, 18]. These studies included between 23 and 1945 patients and used considerably less extensive questionnaires to assess diet quality. Four studies [3, 15, 17, 18] included patients that had been diagnosed for several years. It is likely that these samples entailed more chronic patients than the sample in this study. While bipolar disorder was one of the exclusion criteria of the NESDA study, no CIDI BD was performed at baseline. So, some patients with a history of (hypo)mania might have been included. Yet, overall, the patients with BD in our sample had a relatively short duration of disease, being diagnosed during the 9 years of follow‐up and later in life (mean age between 40 and 50 years), which is generally predictive for a milder course of disease compared to early onset BD [33]. In explorative analyzes in our small BD group, depressive symptoms (e.g., IDS scores) and lifetime (hypo)manic symptoms (e.g., sum of MDQ items) were not correlated to diet quality. Depressive symptom severity, but not lifetime (hypo)manic symptom severity were associated with lower dietary quality in the complete cohort. Previous studies in NESDA showed that symptoms of depression and anxiety were negatively associated with diet scores [34] and found a dose response relationship between severity of the symptoms and dietary quality [22] which was also found in a larger meta‐analysis [21]. It is therefore plausible that the small effect size we found in this cohort is partially due to selection bias.

The more frequent adherence to the Western style diet which was seen in the BD group of the previously mentioned studies, translates to a slightly lower MDS in the current study. The difference in MDS score was found to be driven by a combination of factors within the diet. Patients with BD also had a lower intake of potatoes, fruit, poultry and olive oil, and a higher intake of meat. These dietary factors have been associated previously with a lower risk of severe symptoms of depression [20, 35]. Fruits and unsaturated fats, moreover, have been individually associated with a lower cardiovascular risk, outside the association of the total MDS score, to a more beneficial cardiovascular risk profile [36]. When analyzing the individual subscores, BD patients had a lower intake of potatoes [27] and lower (i.e., unfavorable) MDS scores for ‘alcoholic beverages’. Upon closer inspection however, the MDS score for the latter rates the lowest score for people who consume no alcohol at all. This stems from the idea that a small amount of alcohol is beneficial to health. In the cohort of BD patients, 29% received the ‘worst’ score on this subscore due to complete abstinence. In current views of health management, complete abstinence is considered a positive factor [37]. Limiting alcohol consumption in BD is advised by international guidelines, due to its negative effects on mood, sleep and adherence to pharmacotherapy [38]. This example illustrates that it is still difficult to come to a clear cut conclusion there is still a debate on what should be considered healthy, which is also reflected in the fact that other well‐known and frequently used dietary scores measure the quality of the diet differently [21]. For example, the MDS deducts points for the intake of high fat dairy, while the Dietary Approach to Stop Hypertension (DASH) positively assesses intake of low fat dairy, and the MDS is the only score to include fish and olive oil. Furthermore the DASH makes use of relative scores, awarding points according to which quintile an individual's scores are in [39]. However, most dietary scores agree that a healthy diet is evaluated by positively scoring intake of vegetables, legumes, fruits, wholegrain and negatively scoring processed and red meats [21].

When looking at other covariates, or predictors of general health and cardiovascular risk, BD patients also scored less favorably. They had a higher average BMI than healthy controls. The waist circumference and WHR have been coined to be a better indicator of cardiovascular risk relative to BMI [40, 41]. The waist circumference was also higher on average in BD patients compared to remitted UD patients and healthy controls, but did not differ from patients with current UD. WHR was not statistically significantly different between BD and comparison groups, but showed a similar trend of being higher in patients with BD than in the groups remitted UD and healthy controls. There was no significant difference in WHR between BD patients on antipsychotics and those not using these medications. However, across the entire cohort, antipsychotic users had a significantly higher WHR than non‐users. It is likely that antipsychotics increase visceral fat accumulation through stimulation of appetite, insulin resistance, and other hormonal changes, sleep disturbances, and a lack of physical activity [1, 42].

While the present study has several strengths, including a large number of participants and the use of models controlling for several covariates, there are some limitations that need to be considered. The single FFQ measurement in the cohort limits our ability to infer temporal effects. Our cohort of BD patients consisted of relatively recently diagnosed patients, and the diet quality may be worse with a longer duration of BD. The association between current (hypo)manic symptoms and diet quality could not be analyzed within the BD group. Lifetime (hypo)mania severity was neither correlated to dietary quality in the BD group nor in the complete cohort. Depressive symptom severity was not significantly correlated within this small subsample of 97 participants with BD. Future studies should assess possible associations using (hypo)mania severity, using for example the Young Mania Rating Scale (YMRS) [43] and a larger group of participants with BD for more statistical power. Hypomania may have impaired judgment and memory and led to more irregular intake and meal patterns. It is also conceivable that some BD diagnoses were missed during the baseline assessment since the CIDI BD was not used at baseline. This, however, is unlikely to have caused missed diagnoses since the CIDI BD was used in four measurements after baseline inclusion. NESDA excluded participants with a clinically overt diagnosis of BD at baseline, so our BD group largely comprised incident cases detected during follow‐up. Consequently, individuals with mania/hypomania‐onset BD are likely underrepresented, and findings on diet quality may generalize mainly to those BD patients with depressive‐onset trajectories. Despite these limitations, this study benefits from a larger cohort than many other studies, allowing us to compare dietary quality in patients with BD to several other diagnostic groups.

Previous papers report that less healthy diets, especially when rated with the MDS, are associated with more severe symptoms of depression [20, 22, 44] and increased cardiovascular risks [36, 45]. This last risk is underscored by our finding that BD patients had a higher waist circumference and BMI than healthy controls. Considering the often already present increased cardiovascular risks due to psychotropic use in BD, minimizing other cardiovascular risk factors is important.

To gain further insight, prospective studies with repeated assessments of mood state and diet quality in patients with BD are needed to assess temporal relationships, and there is a need for trials to examine what effects improving diet quality has on mood episodes and physical health in BD. Previously Riedinger et al. published such a study in a small cohort of BD recent‐onset patients (*n* = 39); dietary quality was associated with microbiota diversity, and did not find a significant change in diet quality after 1 year of follow‐up. They advise a higher frequency of measurements for better insights into temporal relationships. There is some preliminary evidence that changing diet might help in the treatment of unipolar depression [46], which might be of use for bipolar depression as well. The constant attention to lifestyle factors that limit cardiovascular risks should be a never wavering element in the treatment of BD.

## 5 Conclusion

In conclusion, dietary quality of patients suffering from BD is of lesser quality than that of people with remitted unipolar depression and of healthy controls, albeit with small effect sizes. Improving dietary habits toward the Mediterranean dietary pattern could offer a non‐invasive and cost‐effective strategy to complement existing treatments for BD, potentially mitigating symptoms and fostering better somatic and mental health outcomes on a population level.

## Funding

This study was funded by the Dutch Research Council (grant no 440.20.009). The infrastructure for the NESDA study is funded through the Geestkracht programme of the Netherlands Organization for Health Research and Development (grant no 10‐000‐1002) and financial contributions by participating universities and mental health‐care organizations (VU University Medical Center, Geestelijke gezondheidszorg (GGZ) inGeest, Leiden University Medical Center, Leiden University, GGZ Rivierduinen, University Medical Center Groningen, University of Groningen, Lentis, GGZ Friesland, GGZ Drenthe, Rob Giel Onderzoekscentrum).

## Conflicts of Interest

The authors declare no conflicts of interest.

## Supporting information


**Table S1:** Links between mood symptoms, food insecurity and financial stress in people with bipolar disorder and in the full cohort.
**Table S2:**. Associations between depressive symptom severity and lifetime (hypo)manic symptom severity on the one hand and dietary quality on the other in BD patients and the total cohort.

## Data Availability

According to European law (General Data Protection Regulation), data containing potentially identifying or sensitive patients' information are restricted. However, for academic researchers, data could be available on request via the NESDA data access committee. See for information on data sharing policy: https://www.nesda.nl/nesda‐english/.
