# Supplementary material for: Dietary Quality in Bipolar Disorder Compared to Unipolar Depression (Current and Remitted) and Healthy Controls: The Netherlands Study of Depression and Anxiety
Source: Bipolar Disord. 2026 Mar 17;28(3):e70104. doi: 10.1111/bdi.70104 (PMC12994118; doi:10.1111/bdi.70104)
Supplement: Supplementary file 1 — Table S1: Links between mood symptoms, food insecurity and financial stress in people with bipolar disorder and in the full cohort. Table S2:. Associations between depressive symptom severity and lifetime (hypo)manic symptom severity on the one hand and dietary quality on the other in BD patients and the total cohort. [file BDI-28-0-s001.docx]

|  | **Lifetime BD (n=100)** | **Current UD (n=199)** | **Remitted UD (n=722)** | **Healthy controls (n=337)** |
| --- | --- | --- | --- | --- |
| **Financial stressors:** |  |  |  |  |
| Food insecurity |  |  |  |  |
| - never | 86 (86.0) | 177 (88.9) | 696 (96.4) | 333 (98.8) |
| - occasionally | 11 (11.0) | 11 (5.5) | 18 (1.5) | 3 (0.9) |
| - regularly/often | 3 (3.0) | 11 (5.5) | 8 (1.1) | 1 (0.3) |
| Money left at the end of the month: |  |  |  |  |
| - usually enough | 59 (59.0) | 99 (49.5) | 457 (63.3) | 266 (78.9) |
| - just enough to make ends meet | 33 (33.0) | 76 (38.2) | 238 (33.0) | 65 (19.3) |
| - insufficient to make ends meet | 8 (8.0) | 24 (12.1) | 27 (3.7) | 6 (1.8) |
| **Sum of MDQ-NL items** |  |  |  |  |
| 0-1 items (n,%) | 26 (26.0) | 78 (39.2) | 425 (58.8) | 271 (80.4)) |
| 2-4 items (n,%) | 31 (31.0) | 78 (39.2) | 198 (27.4) | 45 (11.9) |
| 5-7 items (n,%) | 18 (18.0) | 31 (15.5) | 65 (9.0) | 15 (4.5) |
| 8-13 items (n,%) | 23 (23.0) | 10 (5.0) | 24 (3.4) | 3 (1.2) |
| **IDS-SR score** |  |  |  |  |
| IDS-SR, severity, none (n, %) | 21 (21.0) | 25 (12.6) | 425 (58.9) | 396 (90.8) |
| IDS-SR, severity, mild (n, %) | 29 (29.0) | 63 (31.7) | 217 (30.1) | 27 (8.0) |
| IDS-SR, severity, moderate (n, %) | 28 (28.0) | 72 (36.2) | 65 (9.0) | 2 (0.6) |
| IDS-SR, severity, (very) severe (n, %) | 20 (20.0) | 37 (17.6) | 6 (0.8) | 0 (0) |

**Supplementary tables**

**Supplementary table 1:** Links between mood symptoms, food insecurity and financial stress in people with bipolar disorder and in the full cohort

IDS-SR severity at wave 6 and number of positively scored items (out of 13 total items) on the MDQ-NL. IDS-SR severity none (0-13 points), mild (14-25 points), moderate (26-38), severe and very severe (39 and up). IDS-SR missing 2 for BD, 2 for current UD. MDQ yes missing at wave 6, 2 for BD, 2 for current UD. MDQ yes was missing for 10 participants and IDS for 9 in remitted UD and respectively for 3 and 2 participants in healthy controls.

| **Diet quality (MDS)** | **Standardized beta** | **95% CI** | **p-value** |
| --- | --- | --- | --- |
| **BD cohort (n=97)** |  |  |  |
| Sum of MDQ items | 0.005 | -0.23 ; 0.24 | 0.96 |
| IDS score | -0.004 | -0.23 ; 0.22 | 0.97 |
| **Total cohort (n=1340)** |  |  |  |
| Sum of MDQ items | 0.029 | -0.26 ; 0.08 | 0.30 |
| IDS score | -0.151 | -0.21 ; -0.09 | <0.001 |

**Supplementary table 2**. Associations between depressive symptom severity and lifetime (hypo)manic symptom severity on the one hand and dietary quality on the other in BD patients and the total cohort.

.

Standardized diet quality associated with both IDS-SR and MDQ items adjusted for age, sex and educational level. Missings are listed below supplementary table 1, leading to slightly lower numbers per group than presented in table 1.
